# Gout prevalence and predictors of urate-lowering therapy use: results from a population-based study

**DOI:** 10.1186/s13075-018-1633-9

**Published:** 2018-07-11

**Authors:** Huai Leng Pisaniello, Susan Lester, David Gonzalez-Chica, Nigel Stocks, Marie Longo, Greg R. Sharplin, Eleonora Dal Grande, Tiffany K. Gill, Samuel L. Whittle, Catherine L. Hill

**Affiliations:** 1 Department of Rheumatology, The Queen Elizabeth Hospital, Woodville South, Australia; 2 Discipline of Medicine, Faculty of Health and Medical Sciences, The University of Adelaide, Adelaide, Australia; 3 Discipline of General Practice, Adelaide Medical School, The University of Adelaide, Adelaide, Australia; 4 Drug and Alcohol Services South Australia, Stepney, Australia; 5 Behavioural Research and Evaluation Unit, Cancer Council South Australia, Eastwood, Australia

**Keywords:** Gout, Population, Predictors, Prevalence, Urate-lowering therapy

## Abstract

**Background:**

Gout has an increasing global prevalence. Underutilization of urate-lowering therapy (ULT) is thought to be common, via both suboptimal dosing and poor medication adherence. The aims of this study were to determine the prevalence of self-reported gout and the key predictors of ULT use in those with gout in a representative population survey in South Australia.

**Methods:**

Data were obtained from the Spring 2015 South Australian Health Omnibus Survey, a multilevel, systematic, survey in a representative population sample involving face-to-face interviews (*n* = 3005). This study analyzed responses from respondents aged ≥ 25 years (*n* = 2531) about self-reported gout, ULT use, sociodemographic factors, lifestyle factors, and comorbidities, using survey weighting. Univariate and subsequent adjusted logistic regression analyses on self-reported gout were performed. ULT use was divided into three categories (never use, prior use, and current use) and these data were analyzed using a multinomial logistic regression model.

**Results:**

Self-reported gout prevalence was 6.8% (95% CI 5.8, 7.9). The mean age of respondents with gout was 64 years (standard deviation 16) and 82% were male. As expected, older age, male gender, lower socioeconomic status (SES), and higher body mass index (BMI) were associated with gout, as were high alcohol consumption, current smoking, other forms of arthritis, and hypertension or hypercholesterolemia medication, after adjustment for sociodemographic variables. Two thirds of respondents with gout reported ULT use (36% current; 29% previous) with only 55% continuing treatment. Predictors of ULT use included male gender, low SES, and concomitant cholesterol-lowering therapy. Respondents with gout with a higher BMI were more likely to remain on ULT.

**Conclusions:**

Despite gout being a common, potentially disabling joint disease, only 55% of respondents with gout in this study adhered to ULT. Identification of key predictors of ULT use will provide guidance on prescribing strategy in clinical practice and on the quality of gout care in the community.

**Electronic supplementary material:**

The online version of this article (10.1186/s13075-018-1633-9) contains supplementary material, which is available to authorized users.

## Background

Gout is a chronic inflammatory type of arthritis caused by formation and deposition of monosodium urate crystals in the joints and surrounding tissues [1]. The burden of gout remains substantial with an increasing prevalence estimated in most developed countries, particularly in men and postmenopausal women [2–4]. True estimates of the prevalence of gout have been difficult to determine, with a wide range of estimates depending on the methodology, population and data source. Prevalence of gout in the UK, Germany, Canada, USA, and New Zealand have varied from 1.4% to 3.9% [5–8]. A systematic review of gout prevalence in Australia demonstrated increasing prevalence comparable to that reported in the international data [9]. Within Australia, estimates have varied from 1.5% in a nationwide Australian primary care population study [10] to 5.2% in a recent South Australian population-based cohort study [11].

Current American College of Rheumatology (ACR) and European League Against Rheumatism (EULAR) guidelines emphasize the important roles of patient education and lifestyle modifications, besides pharmacological interventions, for optimal gout management [12, 13]. Effective urate-lowering therapy (ULT) for treatment of chronic gout is widely available and allopurinol as the first-line therapy has been recommended in most international guidelines [12, 13]. Best practice comprises consistent ULT aiming for a target serum urate level determined by comorbidities and the presence of tophi [14].

Despite a good understanding of both pathophysiology and effective treatment, gout remains poorly managed globally with poor treatment adherence [4, 15–17]. In a population-based cohort study using a healthcare register in western Sweden, only one third of patients received ULT within the first year of gout diagnosis and only a quarter of them continued treatment [18]. Interestingly, those who continued treatment were older and had comorbidities, including renal disease [18]. Studies on allopurinol use derived from the Australian community dispensing data have identified suboptimal allopurinol prescribing practice [10] and marked variations in prescribing rates nationwide [19]. However, the trends of ULT prescribing and predictors of use in community settings remain largely unknown. Although both febuxostat and allopurinol are registered in Australia as ULT for gout, febuxostat is restricted to those who have contraindication to or are intolerant of allopurinol or have history of allopurinol hypersensitivity, and therefore, it is seldom used. Therefore, allopurinol use reflects the vast majority of ULT use for gout in Australia.

Many large-cohort international observational studies have demonstrated a strong association between gout and comorbidities such as hypertension, hyperlipidemia, cardiovascular disease, stroke, chronic kidney disease, osteoarthritis, and depression [14, 20]. The 2007–2008 National Health and Nutrition Examination Survey confirmed a high comorbidity prevalence among individuals with gout and hyperuricemia [20]. A case-control study from the UK demonstrated a poorer health status at the time of gout diagnosis compared to matched controls, as well as a higher incident rate of comorbidities [14].

The impact of untreated gout, particularly among those with comorbidities, is substantial, with poor quality of life, less work productivity and increased burden on the healthcare system [21, 22]. Effective gout diagnosis and management in a real-world clinical setting with appropriate initiation and continuation of ULT for this curable inflammatory arthritis is imperative.

The aims of this study were to determine the prevalence of self-reported medically diagnosed gout and the predictors of ULT use in those with gout in a randomly selected population sample in South Australia. In this study, we also assessed the sociodemographic variables, lifestyle factors, and comorbidities relevant to gout, which could affect the two primary outcomes.

## Methods

### Study population

Data were primarily obtained from the South Australian Health Omnibus Survey (SAHOS) during the spring of 2015. However, for comparison of gout prevalence, data were also obtained from the SAHOS conducted during spring 2008.

The SAHOS is an annual, systematic population survey conducted by face-to-face interviews, of approximately 3000 people aged 15 years and over, that obtains cross-sectional representative information on health, wellbeing and related issues among the South Australian population. SAHOS has been designed to meet the highest standards of population survey methodology, and is a clustered, multi-stage, systematic, self-weighting area sample [23, 24]. The spring 2015 SAHOS data consist of 3005 individual face-to-face interviews from 5300 selected households (one individual/household) conducted between September and December 2015, with a participation rate of 66.1%. Data were weighted by the inverse of the individual’s probability of selection, and by the response rate in metropolitan and country regions, and then re-weighted to benchmarks derived from the June 2014 Australian Bureau of Statistics (ABS) estimated resident population for South Australia.

### Data collection

In the survey interview, the respondents were asked “Have you ever been told by a doctor that you have gout?” with the response options of “Yes”, “No”, and “Do not know/refused”. For allopurinol use, the respondents were asked “Do you currently take/have you taken allopurinol for gout?” with the response options of “No, never taken”(“Never”), “No, previously taken”(“Prior”) and “Yes, still taking”(“Current”). A list of the current Australian brand names of allopurinol was given to the respondents. Febuxostat was not included as it only became available for funding in Australia from 2015, and it is only available as a second-line ULT due to its restricted prescribing criteria in Australia.

The SAHOS questions relevant to the current study are outlined in Additional file 1, where each data variable is identified as the actual question number in the survey. Demographic variables (age, gender), socioeconomic status (SES), body mass index (BMI) (derived from questions E1, E2), lifetime risk alcohol consumption (questions I1–I10), smoking history (question S1), fruit and vegetable intake (questions L1, L2), and physical activity (question F1) were assessed.

In detail, SES was determined by the Index of Relative Social Advantage and Disadvantage (IRSAD), which is one of the four postcode-based socioeconomic indexes for areas (SEIFA) developed by the ABS Census data [25]. IRSAD scores were normalized to a mean of 1000 with a standard deviation of 100, with a low score representing the most disadvantaged, and a high score, the most advantaged areas. Self-reported height (cm) and weight (kg) were used to determine the BMI, and categories of BMI were standardized based on the World Health Organization (WHO) criteria [26]. Questions on alcohol consumption were derived from the 2013 National Drug Strategy Household Survey (NDSHS) questionnaire [27], and alcohol lifetime risk was categorized according to the National Health and Medical Research Council (NHMRC) 2009 guidelines [28, 29].

Comorbidities that are relevant for gout, such as cardiovascular disease, diabetes mellitus, hypertension, hypercholesterolemia and other forms of arthritis (questions B1, C1, T1, T2), were determined from physician-diagnosed health condition or medication use. Health-related quality of life (HRQoL) was assessed using the Medical Outcomes Study Short Form 12 (SF-12), a subgroup of 12 questions derived from the original Short Form 36 questionnaire [30]. It has been used internationally and in Australia as a validated discriminatory tool for differentiating groups of disease severity in the general population, and in musculoskeletal conditions [31, 32].

This study survey was approved by the Human Research Ethics Committees of the University of Adelaide (project H-097-2010) and the South Australian Department of Health. The study participation was voluntary with verbal informed consent obtained prior to the interview.

### Statistical analyses

All analyses were performed using appropriate survey weights and were performed using Stata Version 14 (StataCorp 2015; College Station, TX, USA). As gout is most commonly seen in adults and the elderly, only respondents aged 25 years and over were included in the analysis (*n* = 2531).

Individual predictors of gout were analyzed by a series of logistic regression models, both with and without adjustment for sociodemographic variables (gender, and continuous covariates age, BMI, SES). Allopurinol use in respondents with gout was analyzed using a multinomial logistic regression model with three response categories: never use, prior use, and current use. Covariates related to sociodemographic variables (gender, and continuous covariates age, BMI, SES) were automatically included in the model and additional lifestyle, HRQoL, comorbidities, and concomitant medications variables were considered for inclusion in the analysis in a forward step-wise manner (criteria for inclusion *p* > 0.10). The final model was interpreted using Stata post-estimation commands to estimate, for each covariate, population-averaged, marginal probabilities, and dy/dx, which is the change in the predicted population-averaged marginal probabilities of each allopurinol category with a one unit change in the predictor variable. Helmert contrasts of these dy/dx values were used to interpret the results in terms of never versus ever and prior versus current allopurinol use.

## Results

The mean age of the representative population was 52 years (standard deviation, sd 17), the mean BMI was 27.5 (sd 6.1), and 49% were male. In contrast, the mean age of respondents with gout was 64 years (sd 16), the mean BMI was 29.1 (sd 6.3), and 82% were male.

The prevalence of self-reported, medically diagnosed gout in 2015 was 6.8% (95% CI 5.8, 7.9), an increase from a prevalence of 5.8% (95% CI 4.9, 6.8) in 2008 (Table 1, *p* = 0.16). Using direct standardization, the estimated prevalence of gout in 2008, adjusted to the age by gender distribution of the 2015 population sample, was 6.3% (95% CI 5.4, 7.3), indicating a strong consistency in prevalence estimates over time. To enable comparison with other studies, the prevalence of gout by each age-gender stratum in the 2015 data is reported in Additional file 2: Table S1.

**Table 1:** Prevalence (%) of gout (95% confidence intervals), by age and gender, in the South Australian population in 2008 and 2015

Group	2008	2015
Population	5.8 (4.9, 6.8)	6.8 (5.8, 7.9)
Female	3.4 (2.6, 4.5)	2.4 (1.8, 3.2)
Male	8.3 (6.8, 10.1)	11.3 (9.6, 13.4)
25–34 years	1.3 (0.5, 3.1)	0.8 (0.3, 2.6)
35–44 years	2.3 (1.2, 4.3)	2.9 (1.4, 5.8)
45–54 years	3.1 (1.9, 5.0)	6.9 (4.7, 10.0)
55–64 years	8.3 (5.8, 11.6)	7.4 (5.2, 10.3)
65+ years	13.4 (10.9, 16.4)	13.9 (12.0, 16.2)

Although the majority of study respondents were born in Australia (71%), with no further available ethnicity data, respondents with gout were more likely to have been born in the UK (OR 1.6, 95% CI 1.1, 2.5, *p* = 0.022), and less likely to have been born in an Asian country (OR 0.3, 95% CI 0.1, 0.8, *p* = 0.013), Additional file 2: Table S2.

Male gender, older age, lower SES, higher BMI, heavy alcohol use, and current smoking were each associated with gout (Table 2). There was a high prevalence of comorbidities in participants with gout, such as cardiovascular disease (27%), diabetes mellitus (21%), other forms of arthritis (48%), hypertension (59%), and hypercholesterolemia (44%), although only arthritis and concomitant medication use for hypertension and hypercholesterolemia remained independently associated with gout after adjustment for sociodemographic variables (Table 2).

**Table 2:** Sociodemographic and lifestyle variables in the South Australian population, aged 25 years and over, and their relationship with gout (2015 data)

Variable	SA population		Univariate estimate^a^		Adjusted estimate^a^	
	All	With gout	Odds ratio	*p* value	Odds ratio	*p* value
Gender (%)						
Female	51 (49,53)	18 (14,24)	1			
Male	49 (47,51)	82 (76,86)	5.1 (3.7,7.2)	< 0.001		
Age group (%)						
25–34 years	19 (17,22)	2 (1,7)	1			
35–44 years	19 (17,20)	8 (4,15)	3.6 (0.92,14.5)	0.066		
45–54 years	20 (18,22)	20 (14,27)	9.0 (2.6,31.6)	0.001		
55–64 years	18 (16,19)	19 (14,26)	9.7 (2.8,33.7)	< 0.001		
65+ years	25 (23,26)	51 (43,58)	19.8 (6,64.8)	< 0.001		
SES (IRSAD mean)^b^	971 (962,980)	945 (930,961)	0.997 (0.996,0.999)	< 0.001		
BMI (%)						
Normal/underweight	37 (35,39)	23 (17,30)	1			
Overweight	37 (35,39)	40 (33,48)	1.8 (1.2,2.6)			
Obese	26 (24,28)	37 (29,44)	2.3 (1.5,3.6)			
Alcohol lifetime risk (%)						
Abstainers	17 (16,19)	14 (10,20)	1		1	
On average 2 or fewer drinks	64 (62,66)	49 (41,56)	0.9 (0.6,1.5)	0.75	1.0 (0.6,1.6)	0.90
On average more than 2 drinks	19 (17,20)	37 (30,44)	2.6 (1.6,4.3)	< 0.001	2.3 (1.3,4.0)	0.003
Smoking (%)^c^						
Non-smoker	40 (38,43)	26 (2,33)	1		1	
Ex-smoker	44 (42,46)	54 (46,62)	2.0 (1.4,3.0)	< 0.001	1.3 (0.85,2.0)	0.22
Current smoker	16 (14,17)	20 (14,27)	2.1 (1.3,3.3)	0.003	2.0 (1.2,3.6)	0.014
Vegetables ≥ 5 servings/day (%)	7 (6,8)	7 (4,11)	1.0 (0.6,1.8)	0.98	1.0 (0.6,1.9)	0.90
Fruit ≥ 2 servings/day (%)	45 (43,48)	44 (36,51)	0.9 (0.7,1.3)	0.67	1.0 (0.7,1.4)	0.97
Days/week exercise (mean)^d^	3.3 (3.2,3.4)	3.1 (2.7,3.4)	1.0 (0.90,1.0)	0.20	1.0 (0.9,1.1)	0.83
Cardiovascular disease (%)	11 (10,12)	27 (21,34)	3.5 (2.5,5.0)	< 0.001	1.2 (0.8,1.8)	0.43
Diabetes mellitus (%)	10 (9,11)	21 (16,28)	2.7 (1.9,3.8)	< 0.001	1.4 (0.9,2.1)	0.15
Arthritis (any %)	28 (26,28)	48 (41,48)	2.6 (1.9,3.6)	< 0.001	1.7 (1.2,2.5)	0.004
Hypertension on treatment (%)	25 (23,26)	59 (51,66)	5.0 (3.6,6.9)	< 0.001	2.4 (1.6, 3.7)	< 0.001
Hypercholesterolemia on treatment (%)	19 (17,20)	44 (36,51)	3.9 (2.8,5.3)	< 0.001	1.7 (1.2,2.5)	0.004
SF-12 PCS (mean)^e^	50 (47,48)	43.5 (41.7,45.2)	0.96 (0.95, 0.98)	< 0.001	0.99 (0.98,1.01)	0.23
SF-12 MCS (mean)^f^	52 (52,53)	53 (51,54)	1.00 (0.98,1.023)	0.74	0.99 (0.97,1.01)	0.54

Values in brackets represent 95% confidence intervals

*SA* South Australia, *SES* socioeconomic status, *IRSAD* Index of Relative Social Advantage and Disadvantage, *BMI* body mass index, *SF-12* Short Form 12, *PCS* physical component score, *MCS* mental component score

^a^Odds ratios were derived from logistic regression models, with gout as the response variable. All “adjusted” logistic regression models included gender and continuous covariates age, BMI and IRSAD, centred around their mean

^b^Socioeconomic status measured using the IRSAD. The IRSAD is normalized to a mean of 1000 and standard deviation of 100. High scores indicate areas of the most advantage and least disadvantage

^c^The inclusion of alcohol consumption as an additional covariate in the adjusted smoking analysis resulted in a diminution in the odds ratios (ex-smoker, OR 1.2 (95% CI 0.8, 1.9), *p*_diminution_ = 0.051, Current smoker, OR 1.8 (95% CI 1.0, 3.2), *p*_diminution_ = 0.010); however, the direction of the associations remained the same

^d^Exercise was defined as at least 30 min of vigorous activity or 60 min of moderate and/or vigorous activity

^e^SF-12 PCS is normalized to a mean of 50 and a standard deviation of 10

^f^SF12 MCS is normalized to a mean of 50 and a standard deviation of 10

**Fig. 1:**
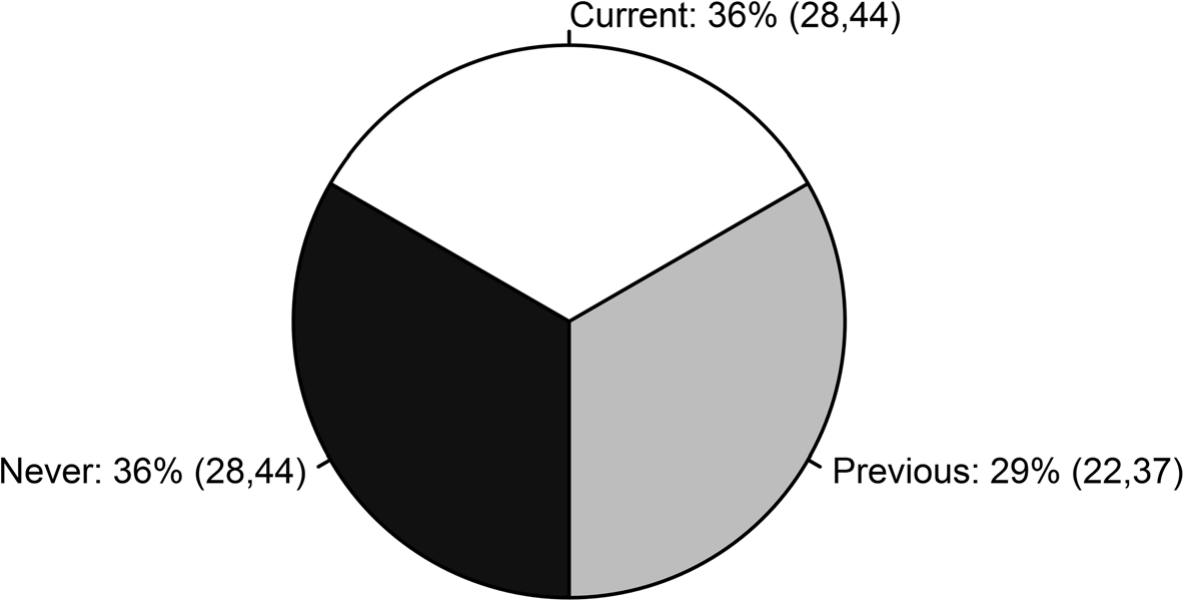
The relative proportions of allopurinol never, previous, and current users among respondents with gout. The brackets enclose 95% confidence intervals

**Fig. 2:**
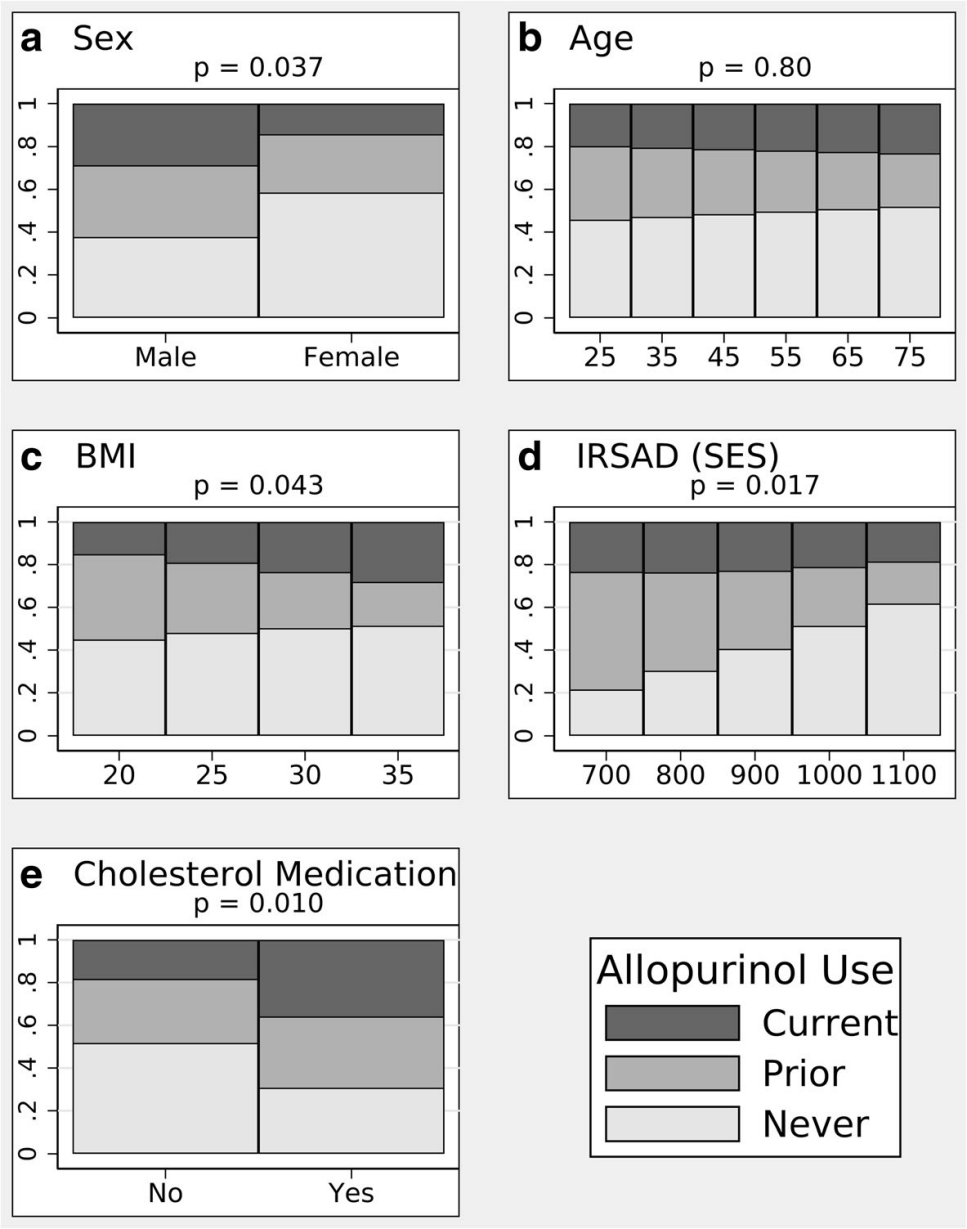
Predicted, population-averaged marginal probabilities of allopurinol use (classified as never, prior, current) estimated from the multinomial regression model, with five predictors as described in “Results”. Representative values were selected for the continuous covariates. **a** Sex. **b** Age. **c** Body mass index (BMI). **d** Index of Relative Social Advantage and Disadvantage (IRSAD) (socioeconomic status (SES)). **e** Cholesterol medication

In this study, the prevalence of current allopurinol use was 36% (95% CI 28, 44) and of previous use was 29% (95% CI 22, 37) (Fig. 1). The final model for predictors of allopurinol use included five covariates: gender, age, BMI, SES, and concomitant medication use for hypercholesterolemia. The regression coefficients for this model are reported in Additional file 2: Table S3A, and information on additional covariates considered, but not included in the final model, is reported in Additional file 2: Table S3B. Results for each covariate were interpreted as population-averaged predicted marginal probabilities for each category of the allopurinol response variable (Fig. 2) and “never versus ever” and “prior vs current” Helmert contrasts of the change in xthese predicted probabilities with a one unit change in the predictor variable (Table 3). Women (*p* = 0.036) and respondents with gout with higher SES status (*p* = 0.006) were less likely to have ever used allopurinol, whereas concomitant cholesterol-lowering medication use was associated with a higher probability of ever use (*p* = 0.004). Higher BMI was the most important predictor of treatment continuation (*p* = 0.015).

**Table 3:** Predictors of allopurinol use among respondents with gout

Predictor	Contrast, dy/dx (95% CI)	*p* value
Female vs male		
Never vs ever	0.312 (0.021, 0.604)	0.036
Prior vs current	0.083 (−0.158, 0.324)	0.50
Joint test		0.037
Age		
Never vs ever	0.002 (− 0.006, 0.010)	0.66
Prior vs current	−0.003 (− 0.011, 0.005)	0.53
Joint test		0.80
BMI		
Never vs ever	0.007 (−0.017, 0.030)	0.57
Prior vs current	−0.022 (− 0.039, − 0.004)	0.015
Joint test		0.043
IRSAD (SES)		
Never vs ever	0.002 (0.000, 0.003)	0.006
Prior vs current	−0.001 (− 0.002, 0.000)	0.15
Joint test		0.017
Cholesterol medication		
Never vs ever	−0.314 (− 0.529, − 0.100)	0.004
Prior vs current	− 0.142 (− 0.435, 0.151)	0.34
Joint test		0.010

Allopurinol use was classified into three categories: “Never”, “Prior”, and “Current”. Analysis was performed by multinomial logistic regression, and interpreted using the change in the predicted population-averaged marginal probabilities of each allopurinol category with a one unit change in the predictor variable (dy/dx). Helmert contrasts of these dy/dx values were used to interpret the results in terms of “Never vs Ever” and “Prior vs Current” allopurinol use

*BMI* body mass index, *IRSAD* Index of Relative Social Advantage and Disadvantage, *SES* socioeconomic status

## Discussion

This 2015 representative population-based study demonstrated a high prevalence of self-reported, medically diagnosed gout (6.8%, 95% CI 5.8, 7.9) in the South Australian population aged 25 and over. The relatively slight increase in gout prevalence since 2008 appears to be attributable to changes in the age-gender distribution of the South Australian population over that time. These estimates of gout prevalence are also comparable to an estimate of 5.2%, in individuals 18 years and over, from the North West Adelaide Health Study cohort weighted to the South Australian estimated resident population of 2009 [11], and are virtually identical to age-standardized estimates from a 1995 Australian population National Health survey [33].

These estimates are likely to accurately reflect the prevalence of gout, over the full spectrum of disease severity, in the South Australian population. Good reliability and sensitivity of self-reported medically diagnosed gout has been validated in a study of two US population-based cohorts (sensitivity of 84% with Cohen’s Κ statistic of 0.63) [34]. Similarly, good reliability of self-reported health risk factors and chronic conditions from computer assisted telephone interview (CATI) surveys has been demonstrated in South Australia [35]. In addition, the question on allopurinol prescribed for gout was appropriately framed for the respondents to correspond if they had been on this medication for gout, and 64% of respondents in this study with self-reported gout had been prescribed allopurinol.

The prevalence of gout in this study is higher than in previous studies from comparable populations. A recent Australian primary-care-based study estimated the national prevalence of gout, ascertained by gout diagnosis or history of allopurinol or colchicine use, as 1.5% [10]. There is likely to be under-ascertainment of gout in the primary-care-based study as it relies on recording of current and previous diagnoses by the general practitioners [10]. Our estimated prevalence is also higher than the prevalence derived from large population-based studies in the UK and Germany (1.4%), Canada (3.8%) and USA (3.9%), but interestingly, relatively similar to the high prevalence in the Maori population and Pacific Islander population (6.1% and 7.6%, respectively) [5–8]. A systematic review of gout prevalence studies identified marked heterogeneity between studies (*I*^2^ = 99.9%), with age, sex, continent, response rate, consistency of data collection, and case definition accounting for the majority of this heterogeneity [36], and a trend for higher prevalence estimates from Australian studies has been previously noted [9]. Some variation in gout prevalence may be specifically attributable to genetic factors, such as variation in renal urate transporter genes [37], particularly SLC2A9 [38, 39] and ABCG2 [40].

We identified expected sociodemographic and lifestyle variables associated with gout, such as older age, male gender, low SES, increased BMI and heavy alcohol consumption. The association between low SES and gout is consistent with previous studies showing a higher prevalence of gout in rural areas in Taiwan, and in less privileged areas in Wales and the northeast of England [4, 41].

Some recent studies have demonstrated an inverse relationship between smoking and gout, leading to the hypothesis that smoking may reduce production of urate from oxidative stress [10, 42–44]. However, we observed a positive association between gout and cigarette smoking, even after adjustment for sociodemographic variables. We had no data on serum urate, and further research to identify the relationships between cigarette smoking, hyperuricemia, and gout is warranted.

There is well-recognized evidence of a high prevalence of comorbidities in the gout population [20]. After adjustment for sociodemographic variables, we observed an association between gout and concomitant medication use for hypertension and hypercholesterolemia, which are the most common comorbidities observed in patients with prevalent gout in most international studies [5, 14, 20, 45]. Other relevant comorbidities, such as cardiovascular disease and diabetes mellitus, although more frequent in respondents with gout, were not independently associated with gout after adjustment for sociodemographic variables.

Gout may be associated with significant pain, activity limitation, and disability and therefore, impact on health-related quality of life (HRQoL), which we assessed in this study using the SF-12 physical and mental components. While the HRQoL physical component was worse in respondents with gout, this did not quite reach accepted levels of statistical significance after adjustment for sociodemographic variables. Nonetheless, these results are consistent with previous studies reporting impaired physical HRQoL [22], worsening function, and impaired work productivity in those with sub-optimally controlled gout [46].

Allopurinol is recommended as the first-line urate-lowering therapy for chronic gout management in most international guidelines [12, 13], and in 2005 it accounted for more than 98% of prescriptions for ULT in Australia [19]. Febuxostat is only available as a second-line ULT in Australia. However, several studies have reported that, in practice, allopurinol use can often be suboptimal [4, 17, 19, 47, 48]. This is the first study to determine the trends and key predictors of ULT use in people with gout from a large representative population-based survey. Approximately two thirds of the respondents with gout (64%) had a history of either previous (29% (95% CI 22, 37%)) or current (36% (95% CI 28, 44%)) allopurinol use, which is comparable to an estimate of 57% from a previous Australian primary-care-based study [10]. Based on a retrospective administrative-claim study in USA on patients with gout, adherence to allopurinol was low with compliance and non-compliance rates of 56% and 44%, respectively [49].

Multiple factors that may be linked to SES, can influence use of and adherence to therapy in chronic diseases such as gout. For example, in the 2008 SAHOS study, at risk or inadequate functional health literacy was identified in more than half of the respondents with gout [50], and adherence to urate-lowering therapy is positively associated with a greater perceived understanding of gout, and inversely associated with perceived severity and consequences of disease [47]. Suboptimal allopurinol adherence has also been reported in the New Zealand Maori compared to the non-Maori population [48]. Therefore, it was somewhat surprising that, in our study, we observed that allopurinol use was less likely in respondents with gout with a higher SES, and that SES did not specifically influence treatment continuity. An explanation for this may be that, because low SES is a risk factor for gout, it may also be an indicator for disease severity, which was not specifically examined in this study. It is also possible that other factors, such as concern about medication use [47], mistrust in its effectiveness [51], or the societal stigma associated with gout [52] may have a disproportionate influence on respondents with gout from a higher SES background.

We also observed an association between cholesterol-lowering medication and allopurinol use. Gout and hyperlipidemia share similar demographic risk factors, and these respondents with gout were perhaps more likely to have been screened for atherosclerosis risk and prescribed cholesterol-lowering medication if warranted.

It is of some concern that, despite evidence that effective management of chronic gout requires treatment with long-term ULT, only 55% of respondents with gout who had ever used allopurinol, remained current users. Our findings are consistent with a systematic review of studies describing ULT use, which reported that on average only 46% of patients with gout were adherent to ULT (assessed by prescription, claims, pill count, and self-report) with non-persistence (non-continuity) ranging between 54 and 87% [53].

We observed that higher BMI was the most important predictor of ULT continuity. Indeed, the role of BMI in the management of gout requires further examination, particularly in the context of an approach of treat-to-urate-target, as it is plausible that patients with gout with a higher BMI may require higher allopurinol doses to achieve urate target. While this has not yet been examined systematically, there is some evidence in support of this hypothesis. A prospective observational study from the US Multiple Risk Factor Intervention Trial (MRFIT) database has demonstrated that among gout patients, a greater than 5% increase in BMI is associated with a 1.6-fold increase in gout attacks compared to those without a significant BMI change [54]. Similarly, a cross-sectional, genetic association study, which classified patients with gout as “good” or “poor” allopurinol responders on the basis of serum urate levels, reported that the poor responders, who received in excess of 300 mg/day allopurinol, had a substantially higher mean BMI [55].

In addition to the use of self-reported, physician-diagnosed gout, there were several other limitations to our study. Data were not available on serum urate levels, or other measures of disease severity, which could better characterize the extent of gout disease in these respondents. The study question on gout treatment was not specifically aimed at any acute gout treatment, and it is acknowledged that gout can be treated with many alternative pharmacologic agents such as probenecid, febuxostat, or colchicine. Furthermore, we did not survey respondents on other urate-lowering agents, as at the time of the survey febuxostat was not subsidized by the Australian national prescribing scheme, and hence, was not widely used. The allopurinol dose and duration of treatment were not included in the interview question and hence, the efficacy of optimal treatment of gout using allopurinol could not be assessed in this survey population.

## Conclusions

This South Australian population-based study has identified a high prevalence of gout and some key predictors of allopurinol use in gout in the community. Despite the importance of ULT for effective gout management, only 55% of respondents with gout who commenced allopurinol remained on it. Low SES was observed in those with gout, which is also a common sociodemographic feature of other chronic diseases, making this an important health-related determinant for policy-makers to consider in our healthcare system planning. Undoubtedly, sociodemographic profile could influence the trajectory of gout treatment. Gout is both an under-appreciated cause of morbidity in the community, and an eminently treatable condition, and further research is required to identify barriers and appropriate clinical management/communication strategies to optimize effective care.

## Additional files

Additional file 1: The spring 2015 SAHOS study questions. (PDF 816 kb)

Additional file 2: **Table S1.** Prevalence of gout by age group and gender from the South Australian 2015 Health Omnibus Survey. **Table S2.** Birth country prevalence (percentage). Odds ratios are for the comparison of participants with and without gout. **Table S3. (**A) Coefficients from the multinomial logistic regression model for predictors of allopurinol use. (B) Predictor variables not included in the model for allopurinol use. (PDF 236 kb)

## Abbreviations

ABS: Australian Bureau of Statistics
ACR: American College of Rheumatology
BMI: Body mass index
CATI: Computer-assisted telephone interview
EULAR: European League Against Rheumatism
HRQoL: Health-related quality of life
IRSAD: Index of Relative Social Advantage and Disadvantage
NDSHS: National Drug Strategy Household Survey
NHMRC: National Health and Medical Research Council
SAHOS: South Australian Health Omnibus Survey
SEIFA: Socioeconomic indexes for areas
SES: Socioeconomic status
SF-12: Short Form 12
ULT: Urate-lowering therapy
WHO: World Health Organization
