# Supplementary material for: Gout prevalence and predictors of urate-lowering therapy use: results from a population-based study
Source: Arthritis Res Ther. 2018 Jul 11;20:143. doi: 10.1186/s13075-018-1633-9 (PMC6042461; doi:10.1186/s13075-018-1633-9)
Supplement: Supplementary file 1 — The spring 2015 SAHOS study questions. (PDF 816 kb) [file 13075_2018_1633_MOESM1_ESM.pdf]

Additional file 1

Additional file: The Spring 2015 SAHOS study questions

GENDER – Male/Female

AGE – year(s)

IRSAD scores: mean scores were calculated, which were determined by SEIFA (data using postcodes available from ABS 2011 Census)

A. SF-12 (Questions A1- A12)

B. ARTHRITIS/OSTEOPOROSIS/GOUT

B1 Have you ever been told by a doctor that you have arthritis? If yes – what type? *Multiple response*

1. Yes – osteoarthritis
2. Yes – rheumatoid
3. Yes – don't know type
4. Yes – other (specify)
5. No

C. DIABETES

C1 Have you ever been told by a doctor that you have diabetes?

1. Yes
2. No

E. HEIGHT/WEIGHT

E1 What is your height without shoes? – centimetres OR feet/inches

E2 What is your weight (undressed in the morning)? – kilograms OR stones/pounds

F. PHYSICAL ACTIVITY

Vigorous activity includes activities like fast cycling, jogging, doing an exercise class, and moderate activity includes things like brisk walking, medium paced swimming, walking to and from work, gardening, doing housework etc.

F1 How many days in the past week have you done any vigorous physical activity for a total of at least 30 minutes, OR any combination of moderate and/or vigorous physical activity for a total of at least 60 minutes? This can include 60 minutes of moderate exercise only.

1. Enter days in the last week (0-7)

I. ALCOHOL

I1 In the last 12 months, how often did you have an alcoholic drink of any kind? *If no longer drink ask if within last 12 months.*

1. Every day
2. 5 to 6 days a week
3. 3 to 4 days a week
4. 1 to 2 days a week
5. 2 to 3 days a month
6. About 1 day a month
7. Less often
8. Not in last 12 months
9. No longer drink
10. Never drunk alcohol

I2 On a day that you have an alcoholic drink, how many standard drinks do you usually have?

1. 20 or more drinks
2. 16-19 drinks
3. 13-15 drinks
4. 11-12 drinks
5. 9-10 drinks
6. 7-8 drinks
7. 5-6 drinks
8. 3-4 drinks

9. 2 drinks
10. 1 drink
11. Half a drink

I3 How often in the last 12 months have you had each of the following number of standard drinks in a day? 20 or more standard drinks a day.
1. Every day
2. 5 to 6 days a week
3. 3 to 4 days a week
4. 1 to 2 days a week
5. 2 to 3 days a month
6. About 1 day a month
7. Less often
8. Never

I4 In the last 12 months how often did you have 11-19 standard drinks a day?
1. Every day
2. 5 to 6 days a week
3. 3 to 4 days a week
4. 1 to 2 days a week
5. 2 to 3 days a month
6. About 1 day a month
7. Less often
8. Never

I5 In the last 12 months how often did you have 7-10 standard drinks a day?
1. Every day
2. 5 to 6 days a week
3. 3 to 4 days a week
4. 1 to 2 days a week
5. 2 to 3 days a month
6. About 1 day a month
7. Less often
8. Never

I6 In the last 12 months how often did you have 5-6 standard drinks a day?
1. Every day
2. 5 to 6 days a week
3. 3 to 4 days a week
4. 1 to 2 days a week
5. 2 to 3 days a month
6. About 1 day a month
7. Less often
8. Never

I7 In the last 12 months how often did you have 3-4 standard drinks a day?
1. Every day
2. 5 to 6 days a week
3. 3 to 4 days a week
4. 1 to 2 days a week
5. 2 to 3 days a month
6. About 1 day a month
7. Less often
8. Never

I8 In the last 12 months how often did you have 1-2 standard drinks a day?

1. Every day
2. 5 to 6 days a week
3. 3 to 4 days a week
4. 1 to 2 days a week
5. 2 to 3 days a month
6. About 1 day a month
7. Less often
8. Never

I9 In the last 12 months how often did you have less than one standard drink per day?

1. Every day
2. 5 to 6 days a week
3. 3 to 4 days a week
4. 1 to 2 days a week
5. 2 to 3 days a month
6. About 1 day a month
7. Less often
8. Never

I10 In the last 12 months how often did you have no alcohol in a day?

1. Every day
2. 5 to 6 days a week
3. 3 to 4 days a week
4. 1 to 2 days a week
5. 2 to 3 days a month
6. About 1 day a month
7. Less often
8. Never

L. DIET/FOOD AND NUTRITION

L1 How many serves of vegetables do you usually eat each day? A serve is equal to half a cup of cooked vegetables or one cup of salad vegetables.

1. Enter number of serves
2. Less than 1 serve
3. None

L2 How many serves of fruit do you usually eat each day? A serve is equal to one medium piece of fruit, or two small pieces of fruit or one cup of diced pieces.

1. Enter number of serves
2. Less than 1 serve
3. None

S. SMOKING

S1 Which of the following best describes your smoking status?

1. I smoke daily
2. I smoke occasionally
3. I don’t smoke now but I used to
4. I’ve tried it a few times but never smoked regularly
5. I’ve never smoked

T. CARDIOVASCULAR DISEASE

T1 Have you ever been told by a doctor that you have any of the following conditions? (multiple response)

1. A heart attack or a heart procedure to unblock vessels in your heart (called angioplasty or stenting)
2. Angina

3. Heart failure
4. Stroke
5. Atrial fibrillation
6. None

T2 Concerning risk factors for cardiovascular (heart) disease…*Multiple response*
1. Are you on medication for high blood pressure?
2. Are you on medication for high cholesterol?
3. If you have diabetes, are you on medication for diabetes?
