# Supplementary material for: Gout prevalence and predictors of urate-lowering therapy use: results from a population-based study
Source: Arthritis Res Ther. 2018 Jul 11;20:143. doi: 10.1186/s13075-018-1633-9 (PMC6042461; doi:10.1186/s13075-018-1633-9)
Supplement: Supplementary file 2 — Table S1. Prevalence of gout by age group and gender from the South Australian 2015 Health Omnibus Survey. Table S2. Birth country prevalence (percentage). Odds ratios are for the comparison of participants with and without gout. Table S3. (A) Coefficients from the multinomial logistic regression model for predictors of allopurinol use. (B) Predictor variables not included in the model for allopurinol use. (PDF 236 kb) [file 13075_2018_1633_MOESM2_ESM.pdf]

Additional file 2

Table S1. Prevalence of gout by age group and gender from the South Australian 2015 Health Omnibus Survey

Gender	Age Group	Gout Prevalence	Std. Err	Lower 95% CI	Upper 95% CI
Male	25-34	0.016	0.010	0.005	0.051
Male	35-44	0.054	0.020	0.026	0.109
Male	45-54	0.107	0.024	0.068	0.165
Male	55-64	0.134	0.025	0.093	0.191
Male	65+	0.237	0.021	0.199	0.280
Female	25-34	0 (no observations)			
Female	35-44	0.004	0.004	0.001	0.030
Female	45-54	0.031	0.011	0.015	0.061
Female	55-64	0.015	0.006	0.007	0.035
Female	65+	0.057	0.009	0.042	0.078

Table S2. Birth country prevalence (%). Odds Ratios are for the comparison of gout to non-gout participants.

Country of Birth	SA Population	Gout population	Odds Ratio (95% CI)	p-val
Australia[1]	70.53%	73.93%	1	
UK & Ireland	10.30%	16.99%	1.6 (1.1, 2.5)	0.022
Europe	6.58%	4.35%	0.6 (0.3, 1.2)	0.16
New Zealand	1.07%	0.92%	0.8 (0.2, 3.4)	0.78
North America	0.42%	0.26%	0.6 (0.1, 4.6)	0.60
Oceania	0.09%	0 (no observations)	NA	
Asia	8.86%	2.61%	0.3 (0.1, 0.8)	0.013
Other	2.16%	0.94%	0.4 (0.1, 3.0)	0.37

[1]1.9% of the SA population born in Australia identified as Aboriginal or Torres Strait Islander compared to 2.1% of participants with gout (p = 0.82)

Table S3A. Coefficients from the multinomial logistic regression model for predictors of allopurinol use.

Allopurinol	Coefficient	Std.Err	95% Lower CI	95% Upper CI	p-val
**Never Taken**	(base outcome)				
**Previously taken**					
Age_64	-0.009	0.015	-0.038	0.019	0.52
BMI_29	-0.055	0.039	-0.132	0.022	0.16
IRSAD_945	-0.005	0.002	-0.010	-0.001	0.011
Female	-0.697	0.511	-1.702	0.307	0.17
Cholesterol medications	0.695	0.418	-0.127	1.517	0.097
constant	-0.362	0.277	-0.906	0.183	0.19
**Currently taken**					
Age_64	0.000	0.015	-0.030	0.030	0.98
BMI_29	0.033	0.038	-0.042	0.108	0.39
IRSAD_945	-0.004	0.002	-0.008	0.001	0.097
Female	-1.237	0.529	-2.276	-0.197	0.020
Cholesterol medications	1.300	0.404	0.507	2.093	0.001
constant	-0.326	0.282	-0.879	0.227	0.25

$F(10,517) = 3.10$, $p = 0.0008$

Table S3B. Predictor variables not included in the model for allopurinol use. Additional variables were screened for inclusion in the model for allopurinol use by adding each predictor variable, in turn, to the model described in Table S2A. Significance was determined from the joint F test of whether the regression coefficients for each predictor variable were equal to zero.

Potential Additional Predictors	F	$df_{numerator}$	$df_{denominator}$	p-val
Alcohol lifetime risk	0.68	4	523	0.60
Smoking	0.69	4	523	0.60
Cardiovascular Disease	0.07	2	525	0.93
Diabetes	0.06	2	525	0.94
Arthritis	1.98	2	525	0.14
Hypertension medications	1.00	2	525	0.37
SF12 physical component score	1.64	2	525	0.20
SF12 mental component score	0.96	2	525	0.38
